# Inflammation and Immunity in Radiation Damage to the Gut Mucosa

**DOI:** 10.1155/2013/123241

**Published:** 2013-03-19

**Authors:** Agnès François, Fabien Milliat, Olivier Guipaud, Marc Benderitter

**Affiliations:** Laboratory of Radiopathology and Experimental Therapeutics, Institute for Radiological Protection and Nuclear Safety, 31 Avenue de la Division Leclerc, 92262 Fontenay-aux-Roses, France

## Abstract

Erythema was observed on the skin of the first patients treated with radiation therapy. It is in particular to reduce this erythema, one feature of tissue inflammation, that prescribed dose to the tumor site started to be fractionated. It is now well known that radiation exposure of normal tissues generates a sustained and apparently uncontrolled inflammatory process. Radiation-induced inflammation is always observed, often described, sometimes partly explained, but still today far from being completely understood. The thing with the gut and especially the gut mucosa is that it is at the frontier between the external milieu and the organism, is in contact with a plethora of commensal and foreign antigens, possesses a dense-associated lymphoid tissue, and is particularly radiation sensitive because of a high mucosal turnover rate. All these characteristics make the gut mucosa a strong responsive organ in terms of radiation-induced immunoinflammation. This paper will focus on what has been observed in the normal gut and what remains to be done concerning the immunoinflammatory response following localized radiation exposure.

## 1. Introduction

The objective of radiation therapy is to deliver a dose of ionizing radiations sufficient to ensure tumor control and to avoid cancer recurrence. The treatment of malignant tumors by radiation therapy is, however, invariably associated with radiation exposure of surrounding healthy tissues and the development of acute injury followed by late structural and/or functional damage, directly or indirectly linked to the initial trauma. Increased tumor control efficiency and life expectancy augment the risk to develop radiation sequels in patients in whom cure has been achieved.

## 2. Pelvic Radiation Therapy and “Pelvic Radiation Disease”

Intestinal tissue is particularly radiosensitive and remains the limiting factor in the application of radiotherapeutic schedules for the treatment of tumors located in the pelvis area. Treatment efficacy relies on a compromise between the quality of tumor control achieved by radiation therapy and the damages generated on intestinal healthy tissues; this is referred to as the benefit/risk ratio [1]. The definition of the irradiation field is governed by several factors that illustrate the necessity to include a proportion of normal tissues: possible tumor extensions undetected by medical imaging techniques, uncertainty concerning patient positioning reproducibility between each fraction, and the mobility of tumor and healthy organs during and between each fraction. Fractionated irradiation reduces the probability of high-dose exposure for mobile parts of the digestive tract, such as the small intestine. Conversely, the risk is increased for fixed segments such as the terminal ileum, colon, or rectum which are often concerned in irradiation protocols for cervical, endometrial, rectal, and prostatic cancers treatments. One strategy to limit normal tissue radiation exposure was to improve radiation therapy techniques and tumor imaging. Thus, precise tumor delineation and the use of three dimensions Conformal Radiation Therapy (3D-CRT) and Intensity-Modulated Radiation Therapy (IM-RT) reduce normal tissue volume located in the irradiation field and consequently treatment side effects.

Radiation gastrointestinal toxicity concerns the majority of patients treated for pelvic cancers. Acute symptoms are declared during or shortly after the end of the radiation therapy and are characterized by abdominal pain, diarrhoea and incontinency, less frequently constipation, bleeding, and mucus discharge. Patient's clinical status sometimes evolves through an aggravation of the acute symptoms, with diarrhoea alternately with constipation, severe abdominal pain, and sometimes nausea and vomiting. Clinical expression of gastrointestinal radiation toxicity often resembles other pathologies such as Crohn's disease, ulcerative colitis, or celiac disease, complicating the diagnosis especially when there has been a long period of time between radiation therapy and clinical symptoms expression. To improve the diagnosis, consideration, and management of the gastrointestinal consequences of radiation therapy of pelvic cancers, ranging from minor changes in bowel habits to severe painful and life-strengthening gastrointestinal dysfunction, the term of “pelvic radiation disease” has been suggested recently by Andreyev et al. [2, 3].

## 3. Radiation-Induced Breakdown of the Fragile Balance Governing Mucosal Homeostasis

The intestine is a hierarchised self-renewing tissue, the entire mucosa being replaced every 3–5 days. Cell production is assumed by stem cells residing at the crypt bottom. The number of stem cells is estimated between 4 to 6 cells per crypt [4]. Daughter cells exit the stem cell compartment, reaching the transit amplifying compartment in which they achieve several divisions and are also named as committed precursors or progenitors cells. New produced cells then migrate along the crypt/villus axis and differentiate into 3 cell lineages of the intestinal mucosa, that is, epithelial bordering cells, goblet cells, and enteroendocrine cells. The fourth cell lineage, the Paneth cells, also derives from stem cells but migrates toward the bottom of the crypts.

Ionizing radiations are used to cure cancers based on their properties to kill cells by energy deposition in tissues, water radiolysis, and production of free radicals damaging DNA, proteins and lipids. Radiation-induced molecular damage on DNA can induce cell phenotypic modifications and/or death by apoptosis, necrosis, or mitotic catastrophe. Indirect biological effects consist in cell water radiolysis and generation of a burst of free radicals responsible for multiple damages to biomolecules, with consequent changes in their structure and functions.

**Figure 1:**
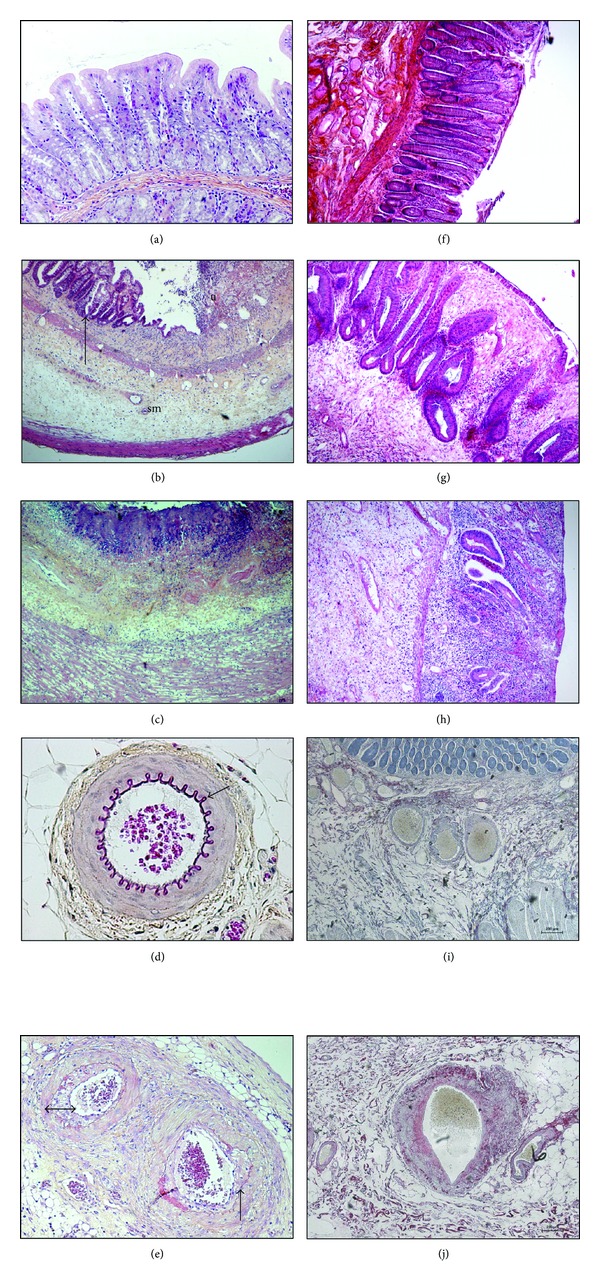
Left panel ((a) to (e)): Radiation-induced damage to the rectal wall following localized exposure to 27 Gy single dose in the rat. (a) Healthy mucosa. (b) Two weeks after exposure, tissue shows mucosal and submucosal inflammation, with mucosal ulceration (u) and submucosal (sm) oedema. Glandular recovering (arrow) alternates with ulcerated areas. (c) Height weeks after exposure, underlying the ulcerated areas, the entire rectal wall is pathologic. The mucosa and submucosa show still severe inflammation, with tissue necrosis at the luminal frontier (purple coloration). Inflammation reaches the oedematous and dystrophic external muscular layers. Note that the muscularis mucosa disappeared. (e) Eight weeks after exposure, severe epithelial, submucosal, and muscular damage is associated with dystrophic submucosal and mesenteric vessels. The elastic layer is dystrophic (arrow) compared to the healthy vessels ((d), arrow) and, neointimal hyperplasia reduces the vessel lumen (double arrow). HeS staining, original magnification ×100 ((a), (d), (e)) or ×40 ((b), (c)), pictures Agnès Francois. Right panel radiation-induced damage to the rectal wall in patients treated for rectal adenocarcinoma, 5 to 7 weeks following the end of radiation therapy (45 Gy). (f) Healthy rectal mucosa. (g): Epithelial atypia with mucosal oedema and inflammation. Crypt positioning is disorganized and some are bifid and show hyperplasia, both signing epithelial regeneration. (h) Severe mucosal ulceration with submucosal oedema and dense inflammatory infiltrate. Crypt number is drastically reduced. (j) Dystrophic submucosal arteriole, with collagen deposition in the vessel wall and neointimal hyperplasia reducing the vascular lumen. (f), (g), (h) HeS staining, (i), (j) Sirius Red staining. Original magnification ×40, pictures Agnès François.

The target cell concept [5, 6] states that tissue response to radiation exposure is governed by cell death in a target radiosensitive compartment, often the stem cell compartment, and that tissue regeneration depends on the surviving and proliferation of stem or progenitor cells. Thus in the case of intestinal mucosa, the severity of radiation damage and its regeneration capacity have long been paralleled to the level of cell death in the stem cell compartment. R-spondin-1, a potent intestinal stem cell growth factor and ligand of LGR5 receptor, protects mice from gastrointestinal syndrome [7, 8] and GLP-2, an intestinal growth factor, reduces both acute and late small intestinal damages following localized high-dose radiation exposure in the rat [9]. However, the increasing knowledge in radiation biology showed that the target cell concept does not reflect what is really happening in the vicinity of irradiated organs. All cell types are sensitive to ionizing radiations, and tissue scaring process initiates immediately after radiation exposure, involving all cell types and compartments of the tissue. Reduction of endothelial cells radiation-induced apottosis by basic fibroblast growth factor protects mice from gastrointestinal syndrome [10], and reduced small intestinal tissue damage following localized radiation exposure in PAI-1 −/− mice is associated with less endothelial cells apoptosis [11]. Tissue response to radiation exposure is considered as a continuum between very acute events and late tissue fibrosis. Conversely to normal scaring, tissue response to ionizing radiation can be considered as a chronic and self-maintained scaring process leading to fibrosis. The acute or prefibrotic phase is characterized by an inflammatory process with tissue damage essentially visible in the mucosal compartment. The young fibrosis shows immunocompetent cells accumulation and mesenchymal cells activation (fibroblasts and smooth muscle cells). Established fibrosis is paucicellular, with densification of scaring tissue and continuous matrix remodelling (Figure 1).

## 4. Radiation-Induced Inflammation

Ionizing radiations can be considered as a proinflammatory signal, and normal tissue response to radiation exposure is immediate and endures with time. Radiation-induced inflammatory response is initiated by the production of reactive oxygen/nitrate species, the induction of apoptosis and clonogenic cell death, mucosal breakdown, and the activation of the transcription of several proinflammatory cytokines, chemokines, and growth factors in the microvascular and mucosal compartments, presumably by recruited immune cells but also by enterocytes and residing cells, depending on the severity of tissue trauma [12, 13]. 

**Figure 2:**
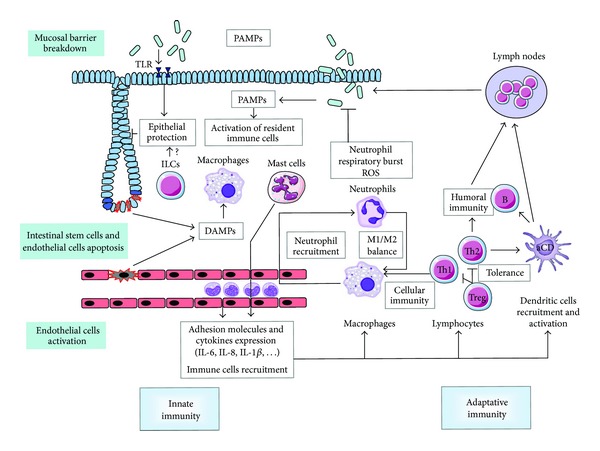
Radiation exposure to the gut mucosa induces epithelial stem cells apoptosis and clonogenic cell death, endothelial cells death and activation, and mucosal barrier breakdown. Innate immunity: resident immune cells such as macrophages and mast cells are activated by DAMPs generated by dead cells and PAMPs coming from mucosal breakdown. Activated endothelial cells express adhesion molecules and cytokines favouring immune cells recruitment into the injured tissue. Activated macrophages increase neutrophil recruitment, which in turn emit signals favouring monocytes recruitment from the blood stream. The radiation-induced tissue M1/M2 balance is unknown. Adaptative immunity: Th1 lymphocytes can activate innate immune cells and favour cell-mediated immunity, whereas Th2 favour humoral immunity via B cells. The balance in irradiated gut tissue is in favour of a Th2 orientation. Treg maintain immune tolerance. Resident and recruited dendritic cells are activated and carry out the link with lymph nodes and the establishment of specific immune response. PAMPs are also detected by TLRs, whose activation can protect epithelium against radiation damage. ILCs play a role in epithelial homeostasis, but their role after radiation exposure is unknown.

The vascular endothelium is a critical target compartment involved in tissue response to radiation exposure and strongly participates in the initiation and development of radiation lesions [5, 14]. Irradiation of the vascular endothelium leads to endothelial cell apoptosis and the acquisition of a proinflammatory, prothrombotic, and antifibrinolytic phenotype, with increased secretion of soluble mediators such as cytokines, chemokines, and growth factors [15]. The increase in adhesion molecules expression such as VCAM-1, ICAM-1, PECAM-1 as well as E and P selectins [16], and the expression of proinflammatory soluble mediators by irradiated endothelial cells activate resident macrophages and favour the early recruitment of polymorphonuclear neutrophils from the bloodstream. Neutrophils are recruited within minutes following tissue trauma, and their presence is characteristic of acute inflammation. Inflammatory process is then amplified by the recruitment and transmigration of monocytes and the activation of resident mast cells, both producing proinflammatory and profibrosing mediators such as IL-1*β*, IL-6, IL-8, CXCL-1, CXCL-2, TNF-*α*, or TGF-*β* [17–20]. The innate immune response carried out by macrophages, neutrophils, and mast cells is supported by the adaptative immune response assumed by the B and T lymphocytes (Figure 2).

### 4.1. Innate or Unspecific Immune Response

Innate immunity insures organisms against pathogens. The first line of defence is the physical barrier carried out by the intestinal mucosa, in which multiple immune cells (macrophages, polymorphonuclear neutrophils, mast cells reside, and innate lymphoid cells) implicated in the immediate unspecific reactions to antigens shared by a plethora of pathogens.

#### 4.1.1. Macrophages

Resident macrophages and circulating monocytes are the sensors of tissue homeostasis breakdown. Upon tissue injury, activated macrophages release a cytokine and chemokine “soup” to recruit neutrophils [21]. Once in the injured tissue, neutrophils can in turn emit signals to favour monocyte recruitment from the bloodstream. Following extravasation, monocytes differentiate into dendritic cells, resident macrophages (M2 type), or inflammatory macrophages (M1 type) depending on the tissue context [22]. Macrophages play a crucial role in the development of inflammation but also in its resolution and tissue regeneration, notably in clearing out tissues from apoptotic neutrophils, bacteria, and cells and tissue debris. The termination of inflammation is orchestrated by many factors. For example, neutrophil apoptosis can trigger several feed-back signals which dampen further neutrophil recruitment, and neutrophil phagocytosis by macrophages can induce in the latters ones a switch in the nature of secreted mediators toward a suppression of the inflammatory response (decreased TNF-alpha and increased TGF-beta and IL-10 secretions) [21].

Fibrotic small bowel of patients with radiation enteritis show altered expression of many genes implicated in stress response, inflammation, and antioxidant metabolism, among them is increased expression of MIP-2, a member of C-X-C chemokine family secreted by monocytes and macrophages and chemoattractive for neutrophils [23]. Rectal biopsies from 33 patients treated with radiotherapy for nongastrointestinal pelvic carcinoma show increased density of macrophages at the end of the second week of treatment mainly in the mucosal compartment [24]. In 17 patients treated for prostate carcinoma, rectal biopsies show increased macrophage invasion 2 and 6 weeks after the commencement of radiotherapeutic treatment [25]. Macrophage immunostaining is increased in the rat small intestine 2 weeks after localized exposure to single dose (21 Gy) or fractionated dose of X-radiation (8 × 5.6 Gy) [26]. Finally, radiation proctitis in mice 2 and 14 weeks following 27 Gy single-dose radiation exposure is associated with increased macrophage density in all tissue compartments [20].

Broadly, authors often report macrophage invasion in irradiated intestine in human tissues as well as in preclinical animal models, but no data exist concerning the putative role played by macrophages in the initiation and development of gut radiation damage. Macrophages, but also neutrophils, are followed in radiation lesions to establish radiation injury scores and put in evidence eventual beneficial effects associated with therapeutic strategies. The understanding of the roles played by macrophages in radiation damage may help to find new therapeutic options. For instance, to favour macrophage M2 phenotype or neutrophil apoptosis may help macrophages to dampen inflammation and would be an interesting therapeutic option but necessitate a refined knowledge of the roles of the different cell types in the successive phases of intestinal tissue response to radiation exposure.

#### 4.1.2. Polymorphonuclear Neutrophils

Neutrophils, with their phagocytic and microbicidal capacities, play a key role in the protection of organisms against infections and participate to the inflammatory response of injured tissues. Radiation-induced intestinal mucosal and vascular barrier breakdown leads to bacterial translocation and to immediate recruitment of neutrophils to the site of injury. The microbicidal properties of the reactive oxygen species (ROS) produced by the so-called neutrophils “respiratory burst” [27] are crucial in the management of the first steps of gastrointestinal infections and inflammation. However, excessive and sustained ROS production may damage healthy cells and tissues and participate in the progression and the chronicity of radiation injury to the intestinal wall. Radiation-damaged tissues, included during the late fibroatrophic phase, often present sustained oxidative stress [28], and the protective effects of probiotics in the pathogenesis of radiation injury to the digestive tract may in part rely on their antioxidant properties [29]. Neutrophil influx is observed in the rat small intestine until 26 weeks after exposure to localized single (18, 21, 29.6 Gy) or fractionated (16 × 4.2 Gy) X-radiation [30, 31]. In mice, radiation proctitis following 27 Gy single-dose exposure shows increased neutrophil numbers in the inflamed and fibrosed areas 2 and 14 weeks postirradiation. The expression of CXCL-1 and CXCL-2, both having a strong neutrophil chemoattractant activity, is increased in irradiated tissues as soon as 3 hours postirradiation, [20]. In humans, biopsies from rectal mucosa of patients at 2 and 6 weeks during the course of ongoing radiotherapy for prostate carcinoma show increased neutrophil numbers [25].

The precise roles of neutrophils in radiation enteritis and proctitis are still unknown and remain ambiguous. Neutrophils are generally considered deleterious in intestinal inflammation. However, in radiation-induced proctitis in mice, increased neutrophil chemoattractant expression and subsequently higher neutrophil numbers were associated with less tissue damage in mast cells-deficient mice [20]. Several studies have reported a potential role of acute neutrophil influx in tissue protection. In *Smad3* knockout mice receiving 30 Gy on the flank skin, reduced tissue radiation damage is associated with increased acute neutrophil influx [32]. In a model of septic shock following cecal ligation and puncture in mice, Alves-Filho et al. noticed that IL-33 favours neutrophils chemotaxis to the site of infection and reduces animal mortality. IL-33-treated animals exhibit enhanced neutrophil influx in the peritoneal cavity and better bacterial clearance [33]. Neutrophil infiltration is a hallmark of irradiated tissues, and several studies are necessary to discriminate between their useful and detrimental properties in the initiation and progression of radiation damage to the digestive tract.

#### 4.1.3. Mast Cells

Mast cells are resident immune cells mainly located in tissues in close contact with the external milieu (gut, lung, and skin) thus participating in innate and adaptative immune reactions [34, 35]. Mast cells are able to secrete a huge number of proinflammatory, profibrosing, vasoactive, and mitogenic soluble mediators (stocked in their granules or newly synthesized) in response to various physiological and pathological situations. Mast cells have been known for a long time to be implicated in allergic reactions but are also involved in several inflammatory and fibrotic disorders [36]. Numerous studies remain necessary to define the precise roles of mast cells in the initiation and development of radiation damage to the intestine. In particular, few data exist, and the diversity of irradiation models generates controversy about mast cells response to irradiation *in vitro* and *in vivo*. Globally, *in vitro*, mast cells are rather radioresistant compared to other immune cells [37]. *In vivo*, mast cells also appear quite radioresistant in mice following 20 Gy fractionated total body irradiation, with no evidence of radiation-induced degranulation [38]. Conversely in the rat, intestinal mast cells are severely depleted after 3.5, 5, or 10 Gy single-dose total body radiation exposure [39, 40]. Human proctitis following radiation therapy for rectal adenocarcinoma is associated with mast cell hyperplasia in the mucosa and submucosa, in muscularis propria, in the dystrophic vessel wall, and more globally in the areas of inflammation and collagen deposition [20]. Mast-cells-deficient rats develop severe acute mucosal damage compared to wild-type animals following localized radiation exposure of the small intestine to 21 Gy-single dose but are protected from late tissue fibrosis [41]. Despite reduced collagen accumulation in fibrotic lesions of mast-cells-deficient rats, TGF*β*-1 mRNA expression in irradiated tissues is similar to their control littermates. Authors suggest that mast cells participate to the profibrosing effect of TGF*β*-1. Experimental radiation proctitis in mice shows mast cells hyperplasia, and mast-cells-deficient mice are protected from both acute (2 weeks) and chronic (14 weeks) rectal damage [20], suggesting again that mast cells play a role in the radiation-induced inflammatory and fibrosing processes in the gut.

Besides a wide range of proinflammatory and profibrosing mediators, mast cells also secrete chymase and tryptase, two proteases implicated in numerous biological processes. Mast cell chymase is involved in vascular, inflammatory, and fibrosing diseases. Chymase can activate matrix metalloproteases, degrades matrix proteins such as fibronectin and vitronectin, and potentiates the biological actions of IL-1*β*, TGF*β*-1, endothelin-1, and angiotensin II [42]. Mast cell tryptase is involved in the regulation of vascular permeability, proliferation, and contraction of smooth muscle cells, angiogenesis, inflammation and fibrosis [43]. Mast cell tryptase can also activate protease-activated receptor-2 (PAR-2). PARs are G-protein-coupled receptors expressed on the surface of many cell types including enterocytes, smooth muscle cells, immune cells, endothelial cells, and stromal cells. PAR-2 is implicated in various biological functions such as intestinal ion transport, motility, vascular tone, inflammation, and cell migration and proliferation in response to various traumas [44]. PAR-2 expression and activation are increased after radiation exposure of the small intestine of the wild-type rat, with significant attenuation in mast cells-deficient rats, showing that deleterious effects of mast cells during the fibroproliferative phase of intestinal radiation injury may partly occur via PAR-2 activation [45, 46]. *In vitro*, exogenously added mast cell chymase or tryptase can turn human colonic muscularis propria smooth muscle cells toward a migrating/proliferating and proinflammatory phenotype, making mast cells putative participants in the *in vivo* radiation-induced inflammatory process and dystrophy of the muscularis propria [20]. Finally, *in vitro* exposure of endothelial cells to mast-cells-conditioned medium exacerbates the radiation-induced overexpression of inflammatory genes such as IL-6, IL-8, CXCL-2, and E-selectin [47], suggesting that mast cells may participate in the activation of vascular endothelium and in the global tissue inflammation following radiation exposure. There is still a lot to be done to understand the roles of mast cells in the response of healthy intestinal tissue to radiation exposure, during the initiation/inflammatory phase of radiation injury and during the recovery process, especially when chronicity takes hold.

#### 4.1.4. Innate Lymphoid Cells (Ilcs)

ILCs regroup various lymphoid cells populations, among which NK cells implicated in the maintenance of epithelial homeostasis and integrity. They produce numerous cytokines and growth factors as potent stimulators of epithelial cell growth [48]. For example, IL-22, expressed by Th cells and ILCs and with a receptor located on stromal mesenchymal and epithelial cells, triggers the production of genes involved in epithelial cell differentiation and survival and has been shown to protect intestinal Paneth and stem cells against immune-mediated tissue damage in a model of graft-versus-host disease in mice. Reciprocally, deficiency in IL-22 led to loss of ILCs and subsequent intestinal barrier disruption and increased tissue damage, showing mutual interactions between immune and epithelial compartments [49]. To our knowledge, no data exist concerning gut ILCs in the development of radiation gut damage following localized exposure.

#### 4.1.5. Pattern Recognition Receptors (PRRs)

Innate response relies on the activation of receptors recognizing pathogens molecular motifs (for Pathogen-Associated Molecular Patterns (PAMPS)) and referred to as Pattern Recognition Receptors (PRRs). Among the different PRRs, Toll-Like Receptors (TLRs) are able to recognize Damage-Associated Molecular Patterns (DAMPs) produced by injured tissues and damaged and activated cells, thus playing a putative role in tissue homeostasis and repair such as postradiation exposure [50]. TLRs are expressed on the surface of multiple cell types such as immune cells, fibroblasts, or intestinal epithelial cells. Pretreatment with the TLR5 receptor ligand flagellin is protective against sublethal doses of whole body irradiation in mice and primates [51] and protects gut mucosal tissue from apoptosis following 8 Gy total body irradiation in mice [52]. In the same way, probiotics inhibit TLR4/NFkB signalling and reduce the severity of experimental colitis [53]. Probiotics also reduce radiation-induced epithelial injury and improve crypt survival in mice following 12 Gy whole body irradiation in a TLR-2/COX-2-dependent manner [54]. There are still no data on the role of TLRs signalling and the development of radiation enteritis and proctitis following high-dose localized intestinal radiation exposure. Working in this direction would warranty interesting breakthroughs and probably open new therapeutic windows to prevent or mitigate severe gut radiation injury.

### 4.2. Adaptative Immune Response

The adaptative immunity consists of immune cell response to specific antigens. This specific response necessitates the presentation to B and T lymphocytes of antigens coming from pathogens by specialized presenting cells such as dendritic cells. The rapid innate response following tissue radiation exposure changes the composition of the microenvironment and favours dendritic cells maturation, antigens presentation and the induction of clonal proliferation of selected immune cells. Adaptative immunity amplifies the first line innate response, so innate and adaptative immunities complement to each other.

Lymphocyte infiltrate is a common feature of irradiated normal tissues, especially in the subacute and chronic inflammatory phases of radiation damage. Following antigen presentation, naïve CD4^+^ T cells can differentiate into different T cells subsets such as Th1, Th2, Th17, or Treg, each showing specific cytokine expression profiles controlled by distinct transcription factors, and assuming various redundant or opposed roles in the tissue immune response to injury.

Immune-modulation properties of ionizing radiation are used in the context of antitumor radiation therapy [55]. In normal tissues, even if lymphocytes subsets have different radiation sensitivities, radiation exposure in total body configuration is used for its immune-suppressive properties. The effects of localized irradiation on lymphocytes are more complex and depend on the irradiated organ, the size of the irradiation field, and the delivery protocol (fraction schedule and total administered dose). CD4^+^ T cells are crucial in the adaptative immune response as they help B-cell to produce antibodies, activate CD8^+^ cytotoxic T cells, and are implicated in the recruitment of innate immune cells such as neutrophils and monocytes/macrophages to the site of tissue damage. CD4^+^ T cells polarisation into Th1, Th2, Th17, and Treg subsets is acquired depending on the tissue cytokine context and is revealed by the activation of specific transcription factors, the expression of several chemokine receptors, a specific cytokine expression profile for each subset, and the activation of preferential partner cells triggering specific immunity [56]. Briefly, Th1 cells are responsible for cell-mediated immunity, stimulate macrophages, and thus contribute to the elimination of intracellular pathogens and promote tumoricidal activity. The Th1 profile is mainly associated with autoimmune diseases. Th2 cells mainly stimulate humoral immunity with B-cell activation and are implicated in allergic reactions. Th17 cells recruit neutrophils and are implicated in the elimination of extracellular pathogens and also participate in the development of autoimmune diseases. Treg cells ensure immunotolerance. Globally, radiation exposure, by its direct effects on adaptative immune cells and by the generation of innate immune response and inflammation, triggers an imbalance in immune populations which still remains obscure [57]. For example, abdominal irradiation in the rat increases Treg populations in the intestine with impaired ability to control effectors T cells that may imbalance immune tolerance, favour inflammation, and compromise normal tissue repair [58]. Still in the rat, fractionated irradiation localized on the colorectum modifies the CD4^+^ T helper polarization through a Th2 profile. The imbalance in favour of Th2 cells persists until 6 months post-irradiation [59]. Th1/Th2 imbalance has been shown to play a determining role in human inflammatory bowel disease, with the development of a Th1 profile in Crohn's disease and a Th2 profile in ulcerative colitis. Continuing immune imbalance may play a significant role in the chronicity of radiation lesions, and further studies are necessary to describe precisely the nature of infiltrating immune cells, to understand the modalities of their recruitment, and finally to highlight the roles played by adaptative immunity in the context of normal digestive tissue response to radiation exposure.

In conclusion, normal gut tissue response to radiation exposure is the result of cell death and activation in all tissue compartments, with a strong oxidative and immunoinflammatory component. The precise roles of the different resident and recruited immune cells described in irradiated normal tissues are still obscure, as well as the part played by innate and adaptative immunities. Strong evidence suggests that ongoing researches in this direction warranty opportunities to discover new therapeutic tools to manage normal tissue radiation damage. Given the relatively poor therapeutic efficiency of “classic” anti-inflammatory strategies, it appears necessary to increase the knowledge concerning enduring oxidative stress, vascular endothelial cell activation, immune cells recruitment and their phenotypic orientations such as M1/M2 macrophages and lymphocytes Th1/Th2/Th17/Treg balances, and finally the conditions necessary to the resolution of radiation-induced inflammation. This may help the understanding of the benefit/risk ratio of the radiation-induced immunoinflammatory response and offer considerable improvement in the benefit of anticancer radiotherapy as well as in the management of normal tissues side effects.
